# Identification of the Prognostic Significance of Somatic Mutation-Derived LncRNA Signatures of Genomic Instability in Lung Adenocarcinoma

**DOI:** 10.3389/fcell.2021.657667

**Published:** 2021-03-29

**Authors:** Wei Geng, Zhilei Lv, Jinshuo Fan, Juanjuan Xu, Kaimin Mao, Zhengrong Yin, Wanlu Qing, Yang Jin

**Affiliations:** ^1^NHC Key Laboratory of Pulmonary Diseases, Department of Respiratory and Critical Care Medicine, Union Hospital, Tongji Medical College, Huazhong University of Science and Technology, Wuhan, China; ^2^Institute of Pathology and Southwest Cancer Center, Southwest Hospital, Third Military Medical University (Army Medical University), Chongqing, China

**Keywords:** genome instability, somatic mutation, lung adenocarcinoma, long non-coding RNA, prognostic signature, survival

## Abstract

**Background:** Lung adenocarcinoma (LUAD) is a highly heterogeneous tumor with substantial somatic mutations and genome instability, which are emerging hallmarks of cancer. Long non-coding RNAs (lncRNAs) are promising cancer biomarkers that are reportedly involved in genomic instability. However, the identification of genome instability-related lncRNAs (GInLncRNAs) and their clinical significance has not been investigated in LUAD.

**Methods:** We determined GInLncRNAs by combining somatic mutation and transcriptome data of 457 patients with LUAD and probed their potential function using co-expression network and Gene Ontology (GO) enrichment analyses. We then filtered GInLncRNAs by Cox regression and LASSO regression to construct a genome instability-related lncRNA signature (GInLncSig). We subsequently evaluated GInLncSig using correlation analyses with mutations, external validation, model comparisons, independent prognostic significance analyses, and clinical stratification analyses. Finally, we established a nomogram for prognosis prediction in patients with LUAD and validated it in the testing set and the entire TCGA dataset.

**Results:** We identified 161 GInLncRNAs, of which seven were screened to develop a prognostic GInLncSig model (LINC01133, LINC01116, LINC01671, FAM83A-AS1, PLAC4, MIR223HG, and AL590226.1). GInLncSig independently predicted the overall survival of patients with LUAD and displayed an improved performance compared to other similar signatures. Furthermore, GInLncSig was related to somatic mutation patterns, suggesting its ability to reflect genome instability in LUAD. Finally, a nomogram comprising the GInLncSig and tumor stage exhibited improved robustness and clinical practicability for predicting patient prognosis.

**Conclusion:** Our study identified a signature for prognostic prediction in LUAD comprising seven lncRNAs associated with genome instability, which may provide a useful indicator for clinical stratification management and treatment decisions for patients with LUAD.

## Introduction

Genome instability and mutations are the enabling characteristics of cancer. Widespread destabilization of the nucleotide sequences is inherent in most human cancers (Hanahan and Weinberg, 2011). Genomic changes occur at different levels, from mutations in single or few nucleotides to gains or losses of entire chromosomes, which may trigger aberrant divisions, multinucleation, and tripolar mitosis (Mackay et al., 2018; S. Zhang et al., 2019). Different cancer types exhibit distinct somatic mutational profiles corresponding to varying numbers of genetic mutations, indicating tissue and cell-specific carcinogenic mechanisms (Lee J. K. et al., 2016; Anandakrishnan et al., 2019). Moreover, as an evolving hallmark of cancer, genomic instability, mainly derived from mutations in DNA repair genes, drives cancer progression and has been identified as a critical prognostic factor (Suzuki et al., 2003; Ottini et al., 2006; Negrini et al., 2010). Therefore, it is of great significance to determine the underlying molecular characteristics of genomic instability in different cancer types and explore their relevant clinical importance.

With the highest morbidity and mortality rates in malignancies, lung cancer is a complex disease characterized by extensive genomic instability (Varella-Garcia, 2010; de Bruin et al., 2014; Bray et al., 2018; Siegel et al., 2020). Risk factors include tobacco smoking, air pollution, and radiation exposure, potentially damaging DNA, resulting in a high rate of genomic alterations (Varella-Garcia, 2010; Dela Cruz et al., 2011). Lung adenocarcinoma (LUAD), the primary subtype of lung cancer, exhibits frequent alterations in proto-oncogenes (e.g., TP53, KRAS, CDKN2A, and STK11), DNA repair defects, and genomic instability (Burgess et al., 2020). Whole-exome sequencing (WES) analysis showed that low genomic instability was associated with better survival in patients with LUAD (Chen Y. et al., 2020). Given that the poor prognosis and clinical heterogeneity of LUAD, developing new biomarkers based on its mutant phenotypes may offer a better read-out for risk stratification and prognostic assessment of patients with LUAD.

It is evident that many genomic mutations in cancer reside in non-coding regions, most of which are further transcribed into transcripts of more than 200 nucleotides, known as long non-coding RNAs (lncRNAs) (Huarte, 2015). In the past decades, increasing evidence has showed that lncRNAs play a critical role in gene regulation, cell proliferation, survival, migration, and genomic stability. These versatile biological functions and their cell- and tissue-specific distribution patterns render them promising cancer biomarkers (Huarte, 2015; Chen et al., 2018b; Statello et al., 2020). Of note, lncRNAs associated with genetic alterations exert a tumor-promoting effect and affect genome instability. For example, a novel lncRNA CCAT2 encompassing the rs6983267 SNP is highly overexpressed in microsatellite-stable colorectal cancer and has neem shown to promote tumor growth, metastasis, and chromosomal instability (Ling et al., 2013). A genome-wide survey assessing somatic copy number alterations (SCNAs) of lncRNAs showed that lncRNAs with high-frequency genomic alterations or residing in focal alteration loci were candidates for carcinogenic lncRNAs (Hu X. et al., 2014). Moreover, cancer-testis lncRNAs reactivated in cancers can promote genome instability and malignant transformation (Qin et al., 2017). Conversely, some lncRNAs, such as NORAD, CUPID1, CUPID2, and DDSR1, promote DNA damage repair and facilitate genome stability (Polo et al., 2012; Lee S. et al., 2016; Betts et al., 2017). Although lncRNAs are critical in regulating genome instability, the clinical significance of genome instability-related lncRNAs (GInLncRNAs) has not been investigated in LUAD. In this study, we identified a group of lncRNA signatures related to genome instability from the genomic and transcriptional levels and probed their prognostic significance in patients with LUAD, with the aim of providing an alternative evaluation of the genome instability-conferred mortality risk of cancer.

## Materials and Methods

### Research Roadmap

**FIGURE 1:**
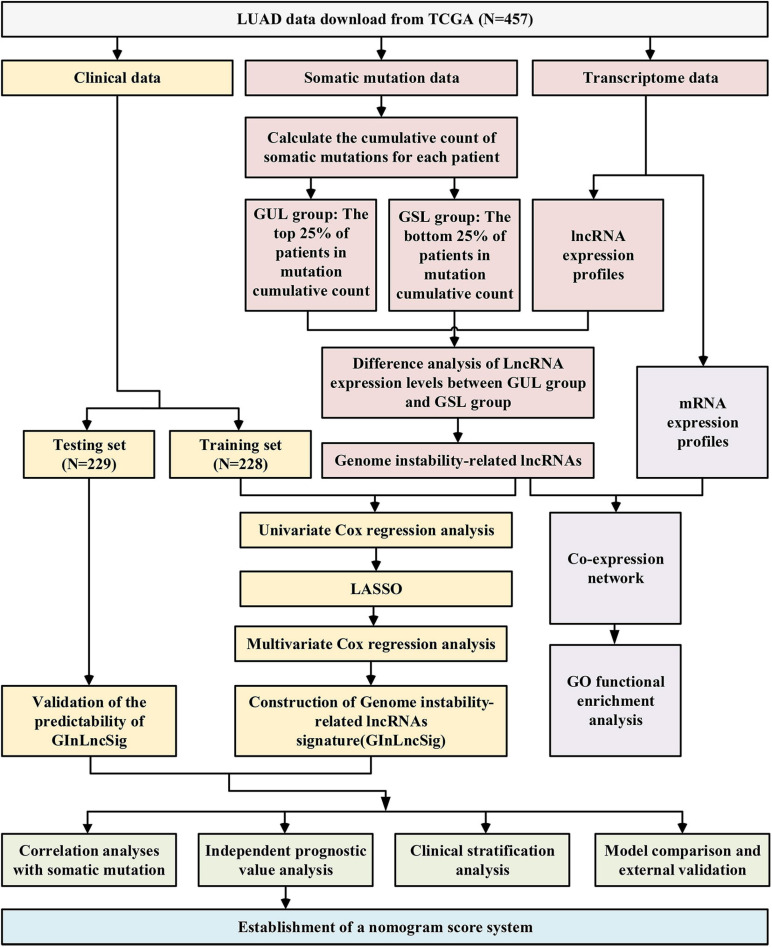
Research roadmap of this study.

The research procedure of this study is depicted in Figure 1. After data collection, GInLncRNAs were identified in combination with somatic mutations and transcriptome data. Co-expression analyses and functional enrichment analyses were conducted to probe the potential function of the above lncRNAs. The patient cohort was then randomly divided into two datasets for training and testing analyses. The GInLncRNAs were further analyzed by Cox regression and LASSO regression to construct a prognostic lncRNA risk signature. The signature was subsequently evaluated using mutation correlation analyses, independent prognostic prediction value analyses, clinical stratification analyses, model comparisons, and external dataset validation. Finally, an optimized model and nomogram were established. The testing set and entire TCGA dataset were subjected to validation of all results.

### Data Collection and Preprocessing

We downloaded the somatic mutation information of 561 patients with LUAD (VarScan version), mRNA and lncRNA transcriptional profiles of 551 patients with LUAD (fragments per kilobase million, FPKM), and clinicopathological features of 486 patients with LUAD from The Cancer Genome Atlas (TCGA) database^1^. We then matched these three parts of the data according to the sample names and removed patients without survival information or with a survival time of less than 30 days to eliminate the interference of non-cancerous causes of death. mRNAs and lncRNAs were annotated using the HUGO Gene Nomenclature Committee (HGNC^2^) database. Finally, a total of 457 samples with complete survival information, somatic mutation data, mRNA and lncRNA expression profiles, and other clinicopathological features were retained for our analysis. To make our study more convincing, we randomly divided the 457 patients into two groups at a ratio of 1:1 using the “caret” package of R software, named as the training set and testing set, respectively. The training set with 229 samples was to identify the genome instability-related lncRNA signature (GInLncSig) and construct a prognostic model. The testing set with 228 samples was used to test the performance of the model. Table 1 shows the clinicopathological characteristics of the cohorts (*P* > 0.05, Chi-squared test).

**TABLE 1:** Clinicopathological information of the patients with LUAD in TCGA cohort.

Covariates	Type	Total (*n* = 457)	Training set (*n* = 229)	Testing set (*n* = 228)	*P* value
Age (%)	≤65	218(47.7%)	109(47.6%)	109(47.81%)	1
	>65	229(50.11%)	114(49.78%)	115(50.44%)	
	Unknown	10(2.19%)	6(2.62%)	4(1.75%)	
Gender (%)	Female	249(54.49%)	124(54.15%)	125(54.82%)	0.9592
	Male	208(45.51%)	105(45.85%)	103(45.18%)	
Tumor Stage (%)	Stage I–II	351(76.81%)	176(76.86%)	175(76.75%)	0.8274
	Stage III–IV	98(21.44%)	51(22.27%)	47(20.61%)	
	Unknown	8(1.75%)	2(0.87%)	6(2.63%)	
T Stage (%)	T1–2	399(87.31%)	195(85.15%)	204(89.47%)	0.1605
	T3–4	55(12.04%)	33(14.41%)	22(9.65%)	
	Unknown	3(0.66%)	1(0.44%)	2(0.88%)	
M Stage (%)	M0	307(67.18%)	156(68.12%)	151(66.23%)	0.2052
	M1	23(5.03%)	8(3.49%)	15(6.58%)	
	Unknown	127(27.79%)	65(28.38%)	62(27.19%)	
N Stage (%)	N0	295(64.55%)	149(65.07%)	146(64.04%)	0.7395
	N1–3	151(33.04%)	73(31.88%)	78(34.21%)	
	Unknown	11(2.41%)	7(3.06%)	4(1.75%)	

*Chi-squared test, *P* < 0.05 means significantly different.*

For external validation, another independent dataset GSE31210 with a large sample size (*N* = 226), basic clinical and survival information, and based on the GPL570 Affymetrix HG-U133_Plus 2.0 platform was retrieved from the Gene Expression Omnibus (GEO) database^3^. Series matrix files containing clinical information and normalized expression profiles of GSE31210 were obtained for our research analysis. We re-annotated the probes of the Affymetrix HG-U133_Plus 2.0 platform into gene symbols by matching the sequence files (HG-U133_Plus_2 Probe Sequences, FASTA format, August 20, 2008) of the probe sets and the annotation files of GENCODE (release 37). The expression levels of probes mapping to the same gene were averaged to obtain a unique value.

### Screening of lncRNAs Related to Genome Instability

We extracted lncRNA expression profiles of samples from the whole annotated transcriptome data and combined them with somatic mutation profiles according to a mutator hypothesis-derived computational workflow (Bao et al., 2020). After computing the cumulative counts of somatic mutations in each sample, we designated the top 25% of the patients and the bottom 25% of the patients having the cumulative number of mutations as genome unstable-like (GUL) group and genome stable-like (GSL) group, respectively. We subsequently compared the mean expression of each lncRNA between the two groups using the Wilcoxon rank-sum test in “limma” package of R software. Consequently, we identified the differentially expressed lncRNAs [|Fold Change| > 1.0 and false discovery rate (FDR) adjusted *P* < 0.05] as ultimate GInLncRNAs. Volcano plot of differentially expressed lncRNAs between GUL group and GSL group was performed using “ggpubr” and “ggthemes” packages of R software.

### Hierarchical Clustering Analyses

We normalized the expression data of GInLncRNAs from all 457 samples using a Z-score analysis. Then we conducted hierarchical clustering analyses with “sparcl,” “pheatmap” and “limma” packages of R software by computing Euclidean distances and cutting the tree into two clusters. The cluster with higher mutation counts was defined as a GU-like cluster, whereas the other was described as a GS-like cluster (*P* < 0.05, Mann–Whitney U test).

### Gene Co-expression Network

We performed the Pearson correlation analysis of the expression levels of lncRNA and mRNA using “limma” package of R software to determine the potential functional mRNA partners co-expressed with GInLncRNAs. The top 10 mRNAs co-expressed with each GInLncRNA were selected according to the Pearson correlation coefficient. We visualized their co-expression network using Cytoscape software and noted the name of GInLncRNAs and their top three co-expressed mRNAs ranked by Pearson correlation coefficient.

### Functional Enrichment Analysis

To identify the possible functions of GInLncRNAs, we carried out Gene Ontology (GO) functional enrichment analysis of their mRNA partners (Chen L. et al., 2017) using “clusterProfiler,” “org.Hs.eg.db,” “enrichplot,” and “ggplot2” packages of R software. Clusters with *P* < 0.05 and *P*.adjust < 0.05 were considered significantly enriched.

### Construction of GInLncRNA-Based Prognostic Signature and Performance Evaluation

First, we conducted univariate Cox regression analysis in the training set using “survival” package of R software to evaluate the relationship between the expression level of GInLncRNAs and patients’ overall survival. The lncRNAs with a Cox *P*-value < 0.05 were considered as candidates with prognostic value. Second, we further filtered candidate GInLncRNAs using the least absolute shrinkage and selection operator (LASSO) regression algorithm with penalty parameter tuning conducted by 10−fold cross−validation with “glmnet” and “survival” packages. Third, the screened GInLncRNAs from LASSO were subjected to stepwise multivariate Cox proportional hazard regression analysis to obtain the optimal candidates and construct a prognostic model of GInLncRNAs. The Receiver Operating Characteristic (ROC) curve analysis was conducted and the Areas Under Curve (AUC) values were obtained to evaluate the prognostic model’s predictability using “survivalROC” package. Then, the GInLncSig for prognosis prediction was developed based on the coefficient of each prognostic GInLncRNAs in the model and their expression levels. The formula for calculating the GInLncSig risk score was as follows:

$$\mathrm{GInLncSig}\mathrm{score}=\sum_{i=1}^{n}coefi\times Xi$$

The “*coefi*” and “*Xi*” represent the coefficient and expression level of each prognostic lncRNAs, respectively. Patients with LUAD were classified into high-risk and low-risk groups based on the median GInLncSig score as the risk cut-off point. The survival curves of the two groups were plotted using the Kaplan–Meier method and compared by the log-rank test using “survival” and “survminer” packages in R with a *p* < 0.05 indicating significance. Finally, the GInLncSig risk model was applied to the testing set and the entire TCGA set to evaluate its performance.

### External Validation and Model Comparison

The GInLncSig model was further validated using another independent GEO cohort of 226 patients profiled using microarray platform. We retrieved the expression of lncRNAs in GInLncSig in the GSE31210 dataset and calculated the risk score of patients based on the aforementioned formula. Patients with LUAD were classified into high-risk and low-risk groups based on the median GInLncSig score. Survival analysis of the two groups were conducted using the Kaplan–Meier method and the log-rank test using “survival” and “survminer” packages in R. *P* < 0.05 indicated statistical significance. Comparison analysis of the expressions of UBQLN4 between two risk groups and correlation analysis between GInLncSig and clinical parameters in GSE31210 dataset were performed using “limma” and “ggpubr” packages. Moreover, we retrieved the LncRNA signature model from other reports and compared their prediction performance by drawing the ROC curve and calculating the AUC values.

### Independent Analysis of GInLncSig in Prognostic Value and Clinical Stratification Analysis

To test whether the GInLncSig is an independent prognostic factor of other key clinicopathological features, we implemented univariate and multivariate Cox regression analyses for each variable in training, testing, TCGA, and GSE31210 datasets using “survival” package of R software. Statistical significance was set at *P* < 0.05. Then, a clinical stratification analysis was conducted further to assess the stability of the prognostic efficacy of GInLncSig. Patients in the whole TCGA set were stratified into subgroups according to clinical parameters, including age (≤65 and >65), gender (female and male), and tumor stage (I-II and III-IV). Patients in each clinical subgroup were further divided into high-risk and low-risk groups based on the median GInLncSig score. Kaplan–Meier analysis and the log-rank test were performed to compare survival differences between the high- and low-risk groups in each subgroup.

### Building and Validation of a Nomogram Score System

Based on multivariate Cox regression analysis in assessing the independent prognostic significance of GInLncSig and clinical variables, we constructed a nomogram in the training set to predict the survival of patients with LUAD. Each variable was allocated a point in the nomogram score system, adding up to a total point for each sample that predicts 1-, 2-, and 3-year survival (Iasonos et al., 2008). The ROC curve, concordance index (C-index), and calibration plot were used to assess the predictive performance and discriminating ability of the nomogram score system. The nonogram was also applied to the testing set and the entire set to verify the above results.

### Statistical Analyses

Chi-squared and Mann–Whitney U test were implemented to explore the differences in categorical and quantitative data between different datasets or groups, respectively. Statistical significance was defined when two-tailed *p* < 0.05. R version 4.0.2 (Institute for Statistics and Mathematics, Vienna, Austria^4^) executed all the statistical analysis and visualization with the corresponding functional package.

## Results

### Screening of Genome Instability-Related lncRNAs in Combination With Somatic Mutation Profiles and Transcriptome Data

**FIGURE 2:**
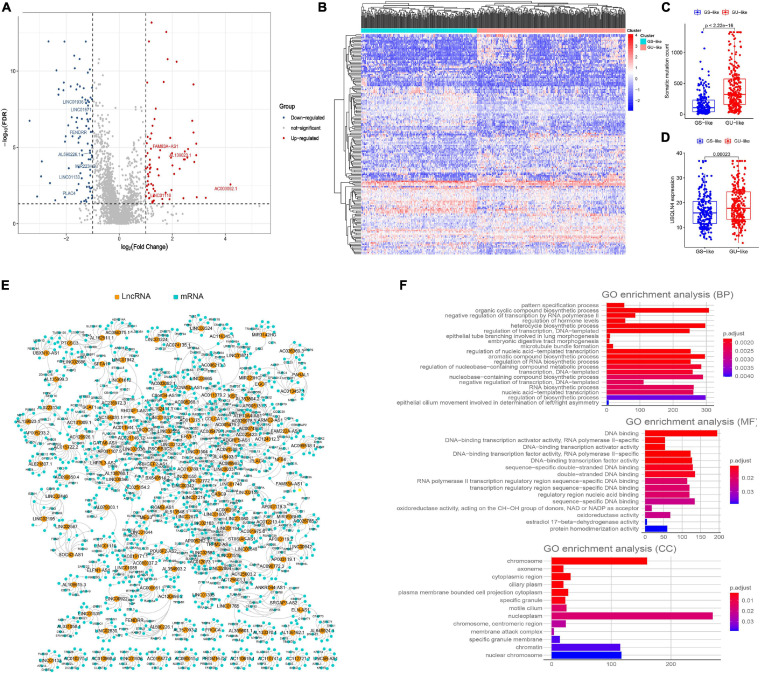
Screening of genomic instability-related lncRNAs and their functional annotation in patients with LUAD. **(A)** Volcano plot of 161 differential expressed lncRNAs between GUL group and GSL group designated as genome instability-related lncRNAs (GInLncRNAs). **(B)** Heatmap of unsupervised hierarchical clustering analyses of 457 patients with LUAD according to the expression levels of 161 GInLncRNAs. The resulting two clusters were determined based on comparing their mean of mutations, with the higher one designated as genome unstable-like (GU-like) cluster (red) and low one termed as genome stable-like (GS-like) cluster (blue). **(C)** Boxplot of comparison of cumulative somatic mutation counts between GU-like cluster and GS-like cluster. The mutation counts of GU-like group were significantly higher than that of the GS-like group (*P* < 0.001, Mann–Whitney U test). **(D)** Boxplot of comparison of the expression levels of UBQLN4 between GU-like cluster and GS-like cluster. UBQLN4 expression levels of the GU-like group were significantly higher than that of the GS-like group (*P* < 0.001, Mann–Whitney U test). **(E)** Network presentation of the relationship between genome instability-related lncRNAs (GInLncRNAs) and their top 10 co-expressed protein-coding genes according to the Pearson correlation coefficient. The orange and blue circles represented the GInLncRNAs and protein-coding mRNAs, respectively. The name of GInlncRNAs and their top three co-expressed mRNAs ranked using Pearson correlation coefficient were plotted in the network. **(F)** Barplot of Go enrichment analyses of the co-expressed protein genes with lncRNAs (*P* < 0.05).

As is shown in Figure 1, we first identified 130 patients with the top 25% of mutations in the cohort as belonging to the GUL group (mean of somatic mutations was 57), and 125 patients with the bottom 25% of mutations in the cohort as belonging to the GSL group (mean of somatic mutations was 539). The clinical information of patients with LUAD in the GSL and GUL groups is depicted in Supplementary Table 1. We found 161 lncRNAs were differentially expressed significantly between the two groups, among which 87 lncRNAs were upregulated, and 74 lncRNAs were downregulated in the GUL group (*P* < 0.05, |logFC| > 1, Figure 2A and Supplementary Table 2).

To determine whether these 161 lncRNAs reflected patients’ genome instability, we applied an unsupervised hierarchical clustering analysis for the expression levels of the 161 lncRNAs in the entire cohort. As shown in Figure 2B, all 457 samples were clustered into two clusters with significantly differential mutation counts, in which the cluster with higher number of mutations was termed the GU-like cluster and that with low number of mutations was termed the GS-like cluster (*P* < 0.05, Mann–Whitney U test; Figure 2C). Moreover, a gene named UBQLN4, which has been reported to drive genomic instability and is overexpressed in aggressive tumors, was also upregulated in the GS-like cluster (*P* < 0.05, Mann–Whitney U test; Figure 2D) (Jachimowicz et al., 2019). These results demonstrated that the 161 lncRNAs could be identified as candidate GInLncRNAs.

We next explored the potential functions of the GInlncRNAs using co-expression analysis with coding genes and the GO functional enrichment analysis. An lncRNA—mRNAs co-expression network that reflected the relationship between the two is displayed in Figure 2E. The name of top three mRNAs co-expressed with each GInlncRNA according to the Pearson correlation coefficient was marked. GO functional enrichment analysis of GInlncRNA-related genes indicated that they were mainly enriched in chromosomes and nucleoplasm in the cellular component (CC), DNA binding in the molecular function (MF), and the transcription and compound synthesis and metabolism in the biological process (BP, *P* < 0.05, Figure 2F and Supplementary Table 3). To prevent DNA damage and to maintain genome stability, exogenous compound synthesis is required to scavenge the excess free radicals or enhance the structural integrity of DNA through binding (Sharma et al., 2020). These results suggested that changes in GInlncRNAs expression may affect genome stability.

### Establishment of a Prognostic Signature Based on Seven Genome Instability-Related lncRNAs and Predictability Evaluation

**FIGURE 3:**
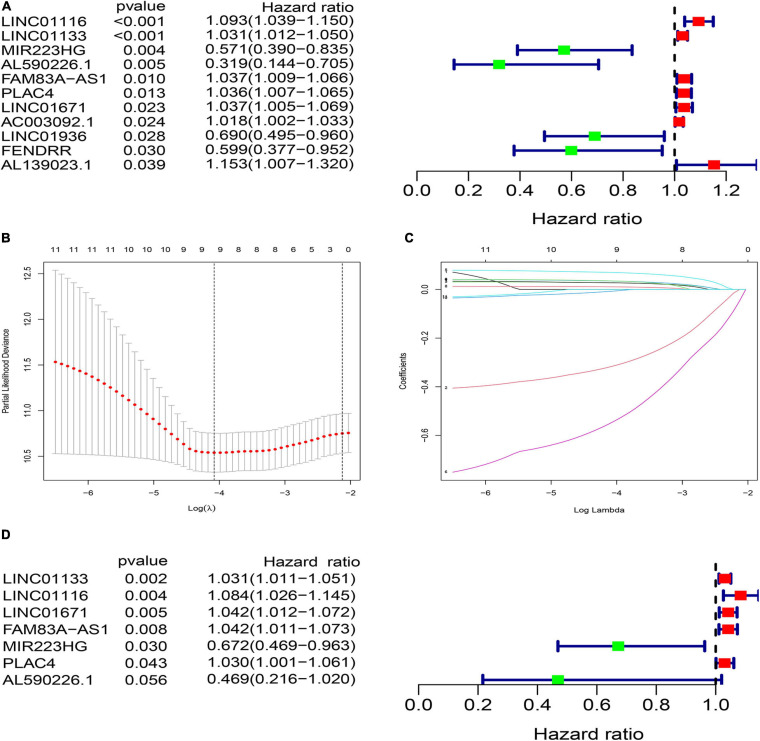
Construction of prognostic risk signature of patients with LUAD using genome instability-related lncRNAs (GInLncRNAs) in the training set. **(A)** Forest plot of eleven GInLncRNAs associated with patients’ overall survival based on univariate Cox regression analyses. Four GInLncRNAs were protecting factors for patients’ survival (MIR223HG, AL590226.1, LINC01936, and FENDRR), while the other seven GInLncRNAs were the risk factors for patients’ survival (LINC01116, LINC01133, FAM83A–AS1, PLAC4, LINC01671, AC003092.1, and AL139023.1). **(B)** The distribution plot of the partial likelihood deviation of the LASSO coefficient. Nine variables were retained when the partial likelihood deviation reached the minimum (Log Lambda = –4.1). **(C)** The distribution plot of the LASSO coefficient. Nine variables were retained when Log Lambda was equal to –4.1. **(D)** The risk signature’s forest plot used seven GInLncRNAs associated with patients’ overall survival (GInLncSig) based on stepwise multivariate Cox proportional hazard regression. Two GInLncRNAs were protecting factors for patients’ survival (MIR223HG and AL590226.1), while the other five GInLncRNAs were the risk factors for patients’ survival (LINC01116, LINC01133, FAM83A–AS1, PLAC4, and LINC01671).

**FIGURE 4:**
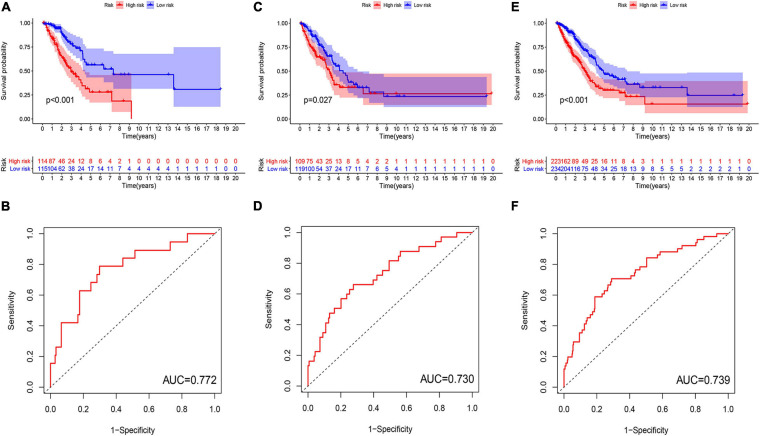
Evaluation and validation of genome instability-related lncRNA signature (GInLncSig)’s predictive performance of overall survival in patients with LUAD in three datasets. Kaplan–Meier survival curves of patients in the high- and low-risk groups separated by GInLncSig score in the training set **(A)**, the testing set **(C)**, and the TCGA set **(E)**. Patients in the low-risk group have prolonged survival than patients in the high-risk group (log-rank test, *P* < 0.05). ROC curves for 1-year survival prediction of the GInLncSig in the training set **(B)**, the testing set **(D)**, and the TCGA set **(F)**.

To explore the clinical significance of GInlncRNAs, we randomly separate the 457 patients into two sets: the training set (*N* = 229) and testing set (*N* = 228), respectively. We then screened 11 lncRNAs significantly associated with the overall survival of patients from the 161 GInlncRNAs in the training set using univariate Cox proportional hazard regression analysis (*P* < 0.05, Figure 3A and Supplementary Table 4). LASSO regression and stepwise multivariate Cox proportional hazard regression analyses were performed to construct a risk model for survival prediction. Consequently, seven of 11 GInlncRNAs that retained prognostic significance (*P* < 0.05) were included in the risk model (Figures 3B–D and Supplementary Table 5). A prognostic signature was then constructed based on the expression levels of seven GInlncRNAs and their coefficients in the multivariate Cox proportional hazard model with the following computational formula: genome instability-related signature (GInLncSig) score = (0.0307 × Expression LINC01133) + (0.0806 × Expression LINC01116) + (0.0409 × Expression LINC01671)+ (0.0408 × Expression FAM83A- AS1) + (0.02998 × Expression PLAC4) + (−0.3974 × Expression MIR223HG) + (−0.7572 × Expression AL590226.1). In the equation of GInLncSig, five lncRNAs (LINC01133, LINC01116, LINC01671, FAM83A-AS1, and PLAC4) have positive coefficient suggesting that they are risk factors and their upregulated expression is associated with poor prognosis, while two lncRNAs (MIR223HG, AL590226.1) with a negative coefficient in the equation worked as protective factors indicating a better survival relevance of their upregulated expression. According to the median GInLncSig score of 1.025, patients with scores higher than the median in the training set were classified as the high-risk group, and those with scores equal to or below the median were classified as the low-risk group (Supplementary Table 6). We found that patients with LUAD in the low-risk group had better survival outcomes than patients in the high-risk group (*P* < 0.001, log-rank test; Figure 4A). The AUC of the ROC curves in the training set was 0.772 for the 1-year survival prediction of GInLncSig (Figure 4B).

To verify the performance of the GInLncSig for survival prediction, we calculated the GInLncSig scores of the testing set and the entire TCGA set and drew their ROC curve. The median GInLncSig score in the testing set was 1.021. Patients in the low-risk group showed a more prolonged survival than patients in the high-risk group with an AUC value of 0.73 in the testing set (*P* = 0.027, log-rank test; Figures 4C,D and Supplementary Table 7). Similar results were also observed in the entire TCGA set, where the AUC of the ROC curves for GInLncSig was 0.739 (*P* < 0.001, log-rank test; Figures 4E,F and Supplementary Table 8). These results indicate that GInLncSig has a good survival prediction efficacy.

### The GInLncSig Was Associated With the Somatic Mutation Pattern

**FIGURE 5:**
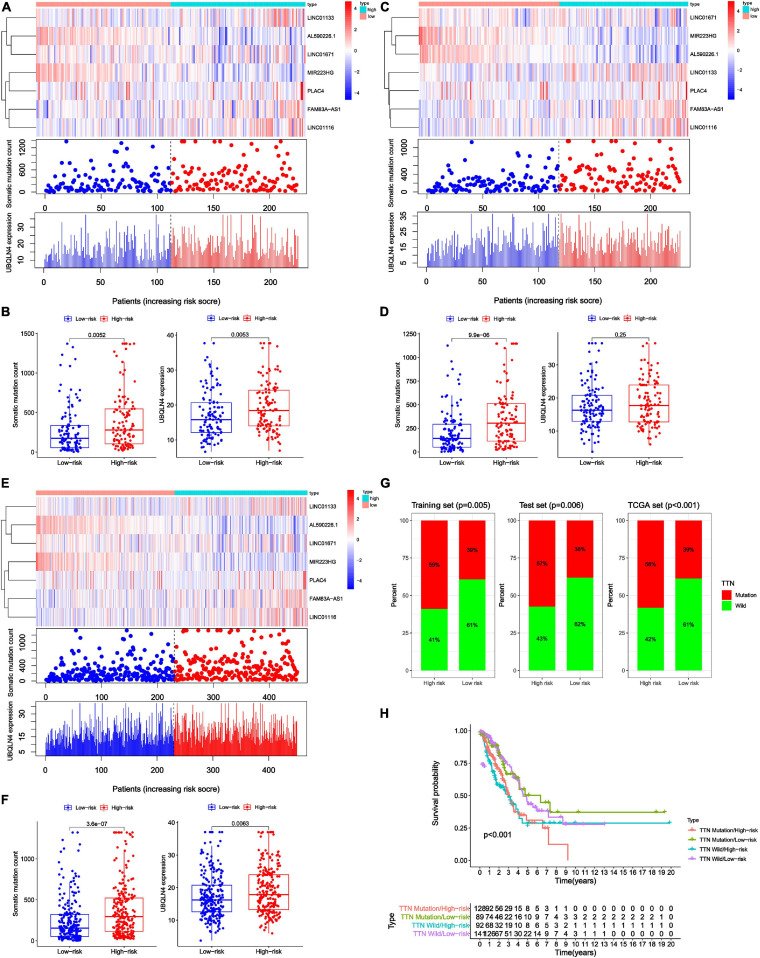
Relationship between genome instability-related lncRNA signature (GInLncSig) and somatic mutation patterns of patients with LUAD in three datasets. **(A,C,E)** A group of risk plots of the training set **(A)**, the testing set **(C)**, and the TCGA set **(E)**, including the heat map of lncRNAs expression, the mutation distribution pattern, and the expression pattern of UBQLN4. The somatic mutation distribution and the expression of lncRNAs and UBQLN4 were changed with the GInLncSig score increasing. **(B,D,F)** Boxplots of comparison of somatic mutation counts and the UBQLN4 expression levels between high- and low-risk groups in the training set [**(B)** *P* < 0.01, Mann–Whitney U test], the testing set [**(D)** *P* < 0.001, Mann–Whitney U test], and the TCGA set [**(F)** *P* < 0.01, Mann–Whitney U test]. **(G)** Boxplots of comparison of the proportion of TTN mutation between the high- and low-risk groups in the training set, the testing set, and the TCGA set (Chi-squared test, *P* < 0.05). **(H)** Kaplan–Meier survival curves of patients in groups divided based on TTN mutation status and the GInLncSig score. The overall survival of the four groups was significantly different (log-rank test, *P* < 0.001).

To test whether GInLncSig is associated with somatic mutation pattern such as the count of somatic mutations and the expression levels of UBQLN4, and the mutation status of the titin gene (TTN). We first performed a group of risk plots for three datasets, including the heat map of lncRNA expression, the distribution of patients’ mutations, and the expression patterns of UBQLN4. As is shown in Figure 5A, the expression levels of LINC01133, LINC01671, LINC01116, FAM83A-AS1, and PLAC4 in the training set increased with the increase in GInLncSig score, whereas the expression of MIR223HG and AL590226.1 decreased with increasing GInLncSig score. Notably, the count of somatic mutations and the expression levels of UBQLN4 also exhibited a growth pattern with the increasing GInLncSig score. As is shown in Figure 5B, the count of somatic mutations in patients in the high-risk group was significantly higher than that of patients in the low-risk group (median ± standard deviation of somatic mutation counts 279.5 ± 373.95 vs. 174 ± 306.58, *P* < 0.01, Mann–Whitney U test; Figure 5B). The expression level of UBQLN4 was significantly higher in the high-risk group than that in the low-risk group (median ± standard deviation of the expression levels of UBQLN4 18.41 ± 7.19 vs. 15.80 ± 6.87, *P* < 0.01, Mann–Whitney U test; Figure 5B). These results were further verified in the testing and the entire TCGA dataset (Figures 5C–F). Similarly, we observed an increasing distribution of somatic mutation counts and the expression of UBQLN4 with increasing GInLncSig scores in both the testing (Figure 5C) and entire TCGA cohort (Figure 5E). Comparison analysis showed that there were significant differences in the number of somatic mutations between the high-risk and low-risk groups in both the testing (median ± standard deviation of somatic mutation counts 304.5 ± 302.86 vs. 143 ± 220.20, *P* < 0.001, Mann–Whitney U test; Figure 5D) and the entire TCGA set (median ± standard deviation of somatic mutation counts 295 ± 347.88 vs. 154.5 ± 314.69, *P* < 0.001, Mann–Whitney U test; Figure 5F). Moreover, we observed a slightly higher expression level of UBQLN4 in the high-risk group than that in the low-risk group in the testing set (median ± standard deviation of the expression levels of UBQLN4 17.71 ± 6.88 vs. 16.23 ± 6.87, *P* = 0.25, Mann–Whitney U test; Figure 5D), and a significantly higher expression level of UBQLN4 in high-risk group than in the low-risk group in the entire TCGA set (median ± standard deviation of the expression levels of UBQLN4 17.84 ± 7.02 vs. 16.23 ± 6.87, *P* < 0.01, Mann–Whitney U test; Figure 5F).

In addition, we observed a high rate of mutation of TTN in our LUAD cohort. Previous studies have reported that somatic mutations in TTN were frequently occur in many cancer types and reflect the status of the tumor mutation burden (Kim et al., 2013; Oh et al., 2020). Therefore, we further assessed the association between the GInLncSig and TTN mutation status. We compared the differences between the high-risk group and low-risk group in three datasets using the chi-square test. The results showed that patients in the high-risk group displayed a significantly higher proportion of TTN mutations than those in the low-risk group among the three datasets (*P* < 0.01, Chi-squared test; Figure 5G). TTN was identified to be associated with platinum resistance in non-small cell lung cancer and prognosis in gastric cancer (Guo et al., 2020; Yang Y. et al., 2020). We further conducted a survival analysis on the risk groups determined using the GInLncSig and the mutation status of TTN, which were TTN Mutaion/ High-risk, TTN Mutaion/ Low-risk, TTN wild/ High-risk, TTN wild / Low-risk groups. As shown in Figure 5H, there was a significant difference among the four groups (*P* < 0.001, log-rank test). These results indicate that the GInLncSig is correlated with TTN mutation status. Taken together, the above results showed that the GInLncSig score was associated with somatic mutation patterns.

### External Validation and Predictability Comparison of the GInLncSig With Other Prognostic lncRNA Signatures

**FIGURE 6:**
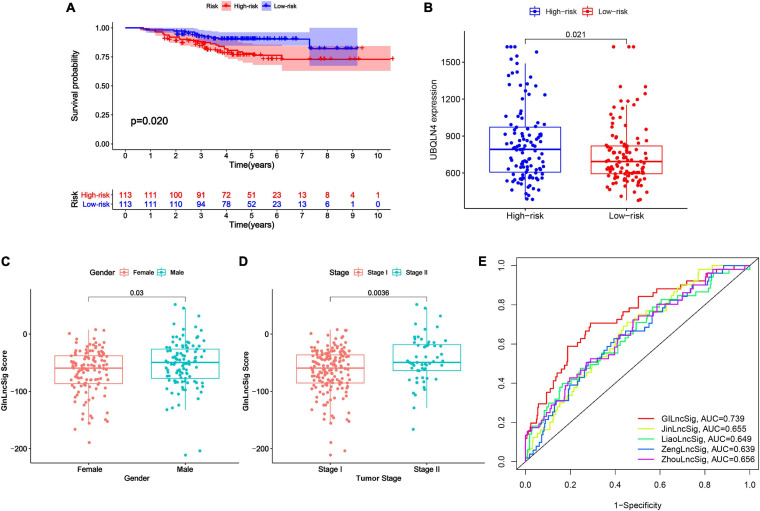
External validation and model comparison. **(A)** Kaplan–Meier survival curves of patients with LUAD in the high- and low-risk groups in GSE31210 dataset. Patients with low GInLncSig score had better survival outcomes than patients with high GInLncSig score (log-rank test, *P* = 0.02). **(B)** Boxplot of comparison of the UBQLN4 expression levels between high- and low-risk groups in GSE31210 dataset (*P* = 0.021, Mann–Whitney U test). **(C)** Boxplot of correlation between GInLncSig score and gender of patients (*P* = 0.03, Mann–Whitney U test). **(D)** Boxplot of correlation between GInLncSig score and tumor stage of patients (*P* = 0.0036, Mann–Whitney U test). **(E)** ROC curves for 1-year survival prediction of the GInLncSig and the other four existing signatures, respectively.

To further validate the prognostic significance of GInLncSig, we investigated the value of GInLncSig in another independent dataset, GSE31210 (*N* = 226), from the GPL570 microarray platform. Although we re-annotated the probes of GPL570 platform, only six of seven lncRNAs in the GInLncSig were covered by the GSE31210 dataset because of the different depths of detection in GPL570 and IlluminaHiSeq platforms. Therefore, we evaluated the significance of GInLncSig scores calculated only based on the expression of six lncRNAs (LINC01133, LINC01116, LINC01671, PLAC4, PLAC4, AP001626.1) according to the aforementioned formula. Survival analysis showed that patients in the low-risk group had a better prognosis than those in the high-risk group (*P* = 0.02, log-rank test; Figure 6A). We also investigated the difference in the expression of UBQLN4 between the two risk groups, and the results showed that the expression level of UBQLN4 in the high-risk group was significantly higher than that in the low-risk group which was consistent with the results of TCGA dataset (*P* = 0.021, Mann–Whitney U test; Figure 6B). Correlation analyses with clinical features demonstrated that the GInLncSig score was associated with gender and tumor stage in patients with LUAD (Figures 6C,D). Male patients tended to have higher GInLncSig scores than female patients (*P* = 0.03, Mann–Whitney U test, Figure 6C). Patients in stage II had significantly higher GInLncSig scores than patients in stage I (*P* = 0.0036, Mann–Whitney U test, Figure 6D). Together, these results further validated the robustness of GInLncSig in LUAD.

Moreover, we also carried out a predictability comparison between GInLncSig and four recently reported lncRNA signatures using the same patients cohort of TCGA for survival prediction of patients with LUAD: thirteen-lncRNA prognostic signature reported by Zhou et al. (2020) (ZhoulncSig), five-lncRNA prognostic signature reported by Zeng et al. (2019) (ZenglncSig), five-lncRNA prognostic signature documented by Liao et al. (2018) (LiaolncSig), and the seven-lncRNA prognostic signature reported by Jin et al. (2020) (JinlncSig). As is depicted in Figure 6E, our GInLncSig with an AUC of ROC for the 1-year OS of 0.739 was more effective in predicting patients’ survival than ZhoulncSig (AUC = 0.656), ZenglncSig (AUC = 0.639), LiaolncSig (AUC = 0.649), and JinlncSig (AUC = 0.655). Together, the above results indicate the credibility and effectiveness of our GInLncSig in predicting the prognosis of patients with LUAD.

### Assessment of Independent Prognostic Significance of GInLncSig and Clinical Stratification Analysis

**FIGURE 7:**
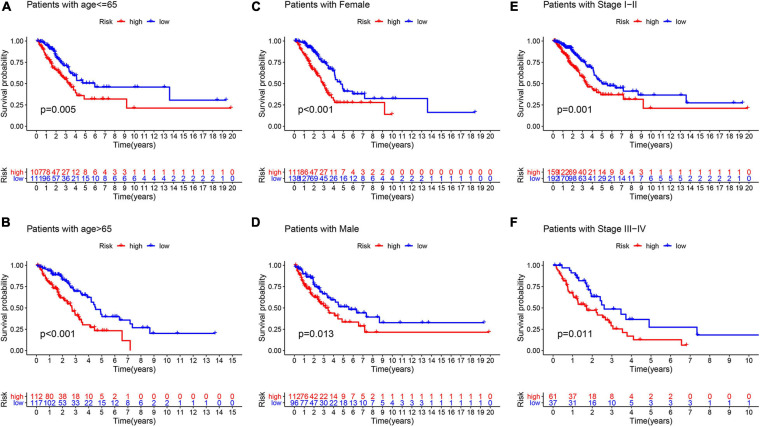
Clinical stratification analysis of the survival difference between patients with LUAD in the high- and low-risk groups by the age, gender, and tumor stage. Kaplan–Meier survival curves of patients in the high- and low-risk groups within six clinically stratified subgroups, including patients with age under 65 years **(A)**, age over 65 **(B)**, the gender of female **(C)**, the gender of male **(D)**, the tumor stage of I–II **(E)**, and the tumor stage of III–IV **(F)**, respectively. Patients in the low-risk group had better survival outcomes than in the high-risk group across all clinically stratified subgroups (log-rank test, *P* < 0.05).

To explore whether GInLncSig is an independent prognostic factor from the clinicopathological features, we implemented univariate and multivariate Cox regression analyses on four datasets (training, testing, TCGA and GSE31210 dataset) for variables including age, gender, tumor stage, and GInLncSig. The results of univariate Cox regression showed that GInLncSig and tumor stage were significantly correlated with the patients’ overall survival in four datasets (*P* < 0.001), and they retained prognostic significance in multivariate Cox regression analyses across four datasets (*P* < 0.01). Other variables, such as age and gender, showed no significant correlation with the patients’ overall survival. Table 2 presents these findings. Clinical stratification analyses of the prognostic performance of GInLncSig in TCGA dataset after adjusted by other clinical factors, including age, gender and tumor stage showed that patients in the low-risk group had better survival outcomes than those in the high-risk group across all clinically stratified subgroups (*P* < 0.05, log-rank test; Figure 7). Together, these results suggested that the prognostic significance of GInLncSig in patients with LUAD is independent of other clinicopathological variables.

**TABLE 2:** Univariate and Multivariate Cox regression analysis of the GInLncSig and clinical features for the independent prognostic significance in four datasets.

Variables	Univariable model				Multivariable model			
	HR	95% CI Lower	95% CI Higher	P-value	HR	95% CI Lower	95% CI Higher	*P*-value
**Training set (*n* = 229)**								
Age	1.013	0.989	1.038	0.291				
Gender	1.291	0.822	2.027	0.267				
Tumor Stage	1.689	1.364	2.092	<0.001	1.643	1.320	2.045	<0.001
GInLncSig	1.196	1.131	1.265	<0.001	1.184	1.118	1.254	<0.001
**Testing set (*n* = 228)**								
Age	1.003	0.983	1.023	0.798				
Gender	1.026	0.678	1.552	0.905				
Tumor Stage	1.593	1.318	1.924	<0.001	1.540	1.270	1.867	<0.001
GInLncSig	1.206	1.131	1.287	<0.001	1.180	1.105	1.261	<0.001
**TCGA set (*n* = 457)**								
Age	1.008	0.992	1.024	0.337				
Gender	1.132	0.836	1.534	0.423				
Tumor Stage	1.634	1.418	1.883	<0.001	1.588	1.374	1.834	<0.001
GInLncSig	1.188	1.142	1.237	<0.001	1.173	1.126	1.222	<0.001
**GSE31210 set (*n* = 226)**								
Age	1.025	0.977	1.075	0.306				
Gender	1.519	0.780	2.955	0.219				
Tumor Stage	4.232	2.175	8.236	<0.001	3.351	1.686	6.660	<0.001
GInLncSig	1.020	1.009	1.031	<0.001	1.016	1.006	1.026	0.002

*HR, hazard ratio; CI, confidence interval.*

### Construction and Validation of a Nomogram for Survival Prediction of Patients With LUAD

**FIGURE 8:**
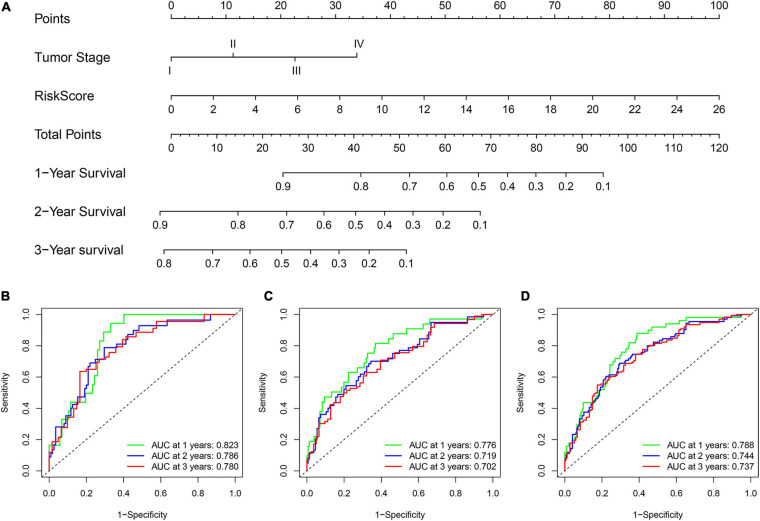
Construction and evaluation of a nomogram for survival prediction of patients with LUAD based on GInLncSig and clinicopathological variables. **(A)** The nomogram developed in training set for predicting 1-, 2-, and 3-year survival of patients. **(B–D)** ROC curves for 1-, 2-, and 3-year survival prediction of the nomogram in the training set **(B)**, testing set **(C)**, and TCGA set **(D)**, respectively.

To improve the model’s clinical practicability, we established a statistical nomogram model in the training set by integrating GInLncSig and tumor stage using “rms” and “survival” packages in R (Figure 8A). The nomogram’s C-index was 0.757, and AUCs of ROC for 1-, 2-, and 3-year survival predictions were 0.823, 0.786, and 0.780, respectively (Figure 8B). Similarly, the C-index was 0.693 in the testing set and the 1-, 2-, and 3-year AUCs were 0.776, 0.719, and 0.702, respectively (Figure 8C). The C-index was 0.720 in the whole TCGA set and the 1-, 2-, and 3-year AUCs were 0.788, 0.744, and 0.737, respectively (Figure 8D). The calibration plot for survival prediction showed good agreement between the actual survival rate and predictions in the three datasets (Supplementary Figure 1). Therefore, these findings indicate improved prediction performance of the nomogram.

## Discussion

Genome instability is a substantial factor that facilitates the acquisition of multiple cancer-related hallmarks (Hanahan and Weinberg, 2011). Constant mutations drive carcinogenesis, tumor progression, and resistance to treatment, endowing the diagnostic and prognostic significance of genomic instability in cancer (Kronenwett et al., 2006; Mettu et al., 2010; Andor et al., 2017). Previous studies have shown that aberrant transcriptional and epigenetic regulation affects genome instability (Ferguson et al., 2015). mRNA and miRNA signatures have been investigated to assess the degree of genome instability in cancer (Habermann et al., 2009; Wang et al., 2017; Chen et al., 2018a). In recent years, lncRNAs, as a promising cancer biomarkers, have also been shown to be involved in genome stability (Qin et al., 2017; Munschauer et al., 2018; Hu W. L. et al., 2018). As a highly heterogeneous disease, lung cancer exhibits a unique genomic profile, with a scattered mutation pattern and widespread somatic mutations (Kandoth et al., 2013). LUAD is the most common carcinoma of the lungs, and no relevant studies have investigated the lncRNA signatures of genome instability in LUAD. Here, we identified a group of GInLncRNAs in LUAD and revealed their significance in predicting patients survival.

In our study, by comparing the expression levels of lncRNAs between patients with differential mutation counts, we first found 161 GInLncRNAs. These lncRNAs were further verified to be associated with genomic instability by hierarchical clustering analyses and following differential analysis of mutation counts and the driver gene of genome instability. We then evaluated the prognostic significance of the 161 GInLncRNAs in patients with LUAD and constructed a GInLncSig consisting of seven lncRNAs (LINC01133, LINC01671, LINC01116, FAM83A-AS1, PLAC4, MIR223HG, and AL590226.1) in the training set. GInLncSig was further proven to be a prognostic factor independent of other clinicopathological characteristics. Patients with a high GInLncSig score tended to have a dismal outcome, which was validated in the testing cohort and GSE31210 dataset. In addition, we observed a significant association between GInLncSig and tumor mutation patterns in LUAD, with a high-risk score being related to high mutations and genome instability. Notably, GInLncSig displayed a robust relationship with prognosis in different clinical subpopulations. Although some effective lncRNA signatures have been developed from GEO microarray datasets for prognosis prediction of lung cancer in recent years, such as the relapse-related lncRNA signature of LUAD (Zhou et al., 2016), tumor immune infiltration-associated lncRNA signature of non-small cell lung cancer (Sun et al., 2020), and the eight prognostic lncRNA signature of non-small cell lung cancer (Zhou et al., 2015). However, we could not conduct a model comparison because the expression profiles from the IlluminaHiSeq platform only covered some of the lncRNAs from the above prognostic signatures. Here, GInLncSig showed an improved prediction performance compared to several existing lncRNA signatures discovered based on the same TCGA cohort. These findings suggest that our GInLncSig is a useful biomarker for predicting patient outcomes and an indicator of genome instability. Given that the tumor stage was also an independent prognostic factor for patients with LUAD in our multivariate cox regression analysis, we finally constructed a nomogram in the training set combining GInLncSig with tumor stage, which further enhanced the prediction model’s comprehension and accuracy. Good performance was further validated in the testing set and the whole TCGA dataset.

Among the seven GInlncRNAs, LINC01133, LINC01671, LINC01116, FAM83A-AS1, and PLAC4 were risk factors for survival, whereas MIR223HG and AL590226.1 were protective factors for patient prognosis. Of note, LINC01133 (Zhang et al., 2015; Zhai et al., 2020; Li et al., 2020; Liu and Xi, 2020; Yang W. et al., 2020), LINC01116 (Cui et al., 2020; Meng et al., 2020), and FAM83A-AS1 (He and Yu, 2019; Shi et al., 2019; Huang et al., 2020) have already been identified to be involved in tumorigenesis and malignant progression in lung cancer or other tumors. For example, Zang et al. (2016) reported that the upregulated expression of LINC01133 in NSCLC was associated with poor patient survival. It can repress KLF2, P21, and E-cadherin transcription through binding to EZH2 and LSD1, thus possessing an oncogenic function in NSCLC (Zang et al., 2016). Zeng et al. (2020) found that LINC01116, overexpressed in LUAD, promoted tumor proliferation and metastasis. Xiao et al. (2019) revealed that FAM83A-AS1 accelerated tumor migration and invasion by targeting miR-150-5p and modifying MMP14 in LUAD. PLAC4, located in 21q22.2, is documented highly expressed in the placenta, and SNPs in the transcriptional regions are associated with fetal trisomy 21 (Lo et al., 2007). The other three lncRNAs, LINC01671, MIR223HG, and AL590226.1, were first reported in our study. The mechanisms of their function in LUAD require further investigation.

This study has several limitations. First, although we investigated the potential value of GInLncSig using bioinformatics analysis and conducted external validation using another independent GEO dataset, experimental validation of our lncRNA signature it still lacking. Therefore, further studies are warranted. Second, the four genomic instability-related lncRNAs (LINC01671, MIR223HG, AL590226.1, and PLAC4) were first reported to be associated with LUAD prognosis, and further investigation is required to clarify their mechanism in carcinogensis and progression of LUAD.

## Conclusion

In summary, our study identified a risk prognostic signature comprising seven genomic instability-related lncRNAs. The GInLncSig could predict the overall survival of patients with LUAD and indicate genomic instability. Moreover, we achieved an improved predictive performance by combining GInLncSig with the tumor stage to construct a nomogram. This is the first study to investigate lncRNA signatures as genomic instability-related biomarkers for predicting the survival of patients with LUAD. Our study may provide a useful indicator for clinical stratification management and treatment decisions for patients with LUAD and a cornerstone for future mechanistic studies of their relationship.

## Data Availability Statement

Publicly available datasets were analyzed in this study. This data can be found here: https://portal.gdc.cancer.gov/, https://www.ncbi.nlm.nih.gov/geo/query/acc.cgi.

## Author Contributions

YJ designed the research framework and revised the manuscript. WG, ZL, JF, JX, and KM contributed to the data analysis. WG and ZL wrote the manuscript. ZY and WQ provided comments during the writing. All authors reviewed and approved the final manuscript.

## Conflict of Interest

The authors declare that the research was conducted in the absence of any commercial or financial relationships that could be construed as a potential conflict of interest.

## Footnotes

1 https://portal.gdc.cancer.gov/

2 http://www.genenames.org/

3 https://www.ncbi.nlm.nih.gov/geo/query/acc.cgi

4 https://www.r-project.org

## References

[B1] Anandakrishnan R. Varghese R. T. Kinney N. A. Garner H. R. (2019). Estimating the number of genetic mutations (hits) required for carcinogenesis based on the distribution of somatic mutations. *PLoS Comput. Biol.* 15:e1006881. 10.1371/journal.pcbi.1006881 30845172 PMC6424461

[B2] Andor N. Maley C. C. Ji H. P. (2017). Genomic instability in cancer: teetering on the limit of tolerance. *Cancer Res.* 77 2179–2185. 10.1158/0008-5472.CAN-16-1553 28432052 PMC5413432

[B3] Bao S. Zhao H. Yuan J. Fan D. Zhang Z. Su J. (2020). Computational identification of mutator-derived lncRNA signatures of genome instability for improving the clinical outcome of cancers: a case study in breast cancer. *Brief Bioinform.* 21 1742–1755. 10.1093/bib/bbz118 31665214

[B4] Betts J. A. Moradi Marjaneh M. Al-Ejeh F. Lim Y. C. Shi W. Sivakumaran H. (2017). Long noncoding RNAs CUPID1 and CUPID2 mediate breast cancer risk at 11q13 by modulating the response to DNA damage. *Am. J. Hum. Genet.* 101 255–266. 10.1016/j.ajhg.2017.07.007 28777932 PMC5544418

[B5] Bray F. Ferlay J. Soerjomataram I. Siegel R. L. Torre L. A. Jemal A. (2018). Global cancer statistics 2018: GLOBOCAN estimates of incidence and mortality worldwide for 36 cancers in 185 countries. *CA Cancer J. Clin.* 68 394–424. 10.3322/caac.21492 30207593

[B6] Burgess J. T. Rose M. Boucher D. Plowman J. Molloy C. Fisher M. (2020). The therapeutic potential of DNA damage repair pathways and genomic stability in lung cancer. *Front. Oncol.* 10:1256. 10.3389/fonc.2020.01256 32850380 PMC7399071

[B7] Chen L. Pan X. Hu X. Zhang Y. H. Wang S. Huang T. (2018a). Gene expression differences among different MSI statuses in colorectal cancer. *Int. J. Cancer* 143 1731–1740. 10.1002/ijc.31554 29696646

[B8] Chen L. Zhang Y. H. Lu G. Huang T. Cai Y. D. (2017). Analysis of cancer-related lncRNAs using gene ontology and KEGG pathways. *Artif. Intell. Med.* 76 27–36. 10.1016/j.artmed.2017.02.001 28363286

[B9] Chen L. Zhang Y. H. Pan X. Liu M. Wang S. Huang T. (2018b). Tissue expression difference between mRNAs and lncRNAs. *Int. J. Mol. Sci.* 19:3416. 10.3390/ijms19113416 30384456 PMC6274976

[B10] Chen Y. Tang W. F. Lin H. Bao H. Li W. Wang A. (2020). Wait-and-see treatment strategy could be considered for lung adenocarcinoma with special pleural dissemination lesions, and low genomic instability correlates with better survival. *Ann. Surg. Oncol.* 27 3808–3818. 10.1245/s10434-020-08400-1 32239339

[B11] Cui L. Chen S. Wang D. Yang Q. (2020). LINC01116 promotes proliferation and migration of endometrial stromal cells by targeting FOXP1 via sponging miR-9-5p in endometriosis. *J. Cell Mol. Med*. 25 2000–2012. 10.1111/jcmm.16039 33372387 PMC7882988

[B12] de Bruin E. C. McGranahan N. Mitter R. Salm M. Wedge D. C. Yates L. (2014). Spatial and temporal diversity in genomic instability processes defines lung cancer evolution. *Science* 346 251–256. 10.1126/science.1253462 25301630 PMC4636050

[B13] Dela Cruz C. S. Tanoue L. T. Matthay R. A. (2011). Lung cancer: epidemiology, etiology, and prevention. *Clin. Chest. Med.* 32 605–644. 10.1016/j.ccm.2011.09.001 22054876 PMC3864624

[B14] Ferguson L. R. Chen H. Collins A. R. Connell M. Damia G. Dasgupta S. (2015). Genomic instability in human cancer: molecular insights and opportunities for therapeutic attack and prevention through diet and nutrition. *Semin. Cancer Biol.* 35(Suppl) S5–S24. 10.1016/j.semcancer.2015.03.005 25869442 PMC4600419

[B15] Guo A. X. Xiao F. Shao W. H. Zhan Y. Zhang L. Xiong J. (2020). Sequential whole exome sequencing reveals somatic mutations associated with platinum response in NSCLC. *Onco Targets Ther.* 13 6485–6496. 10.2147/OTT.S254747 32753889 PMC7342605

[B16] Habermann J. K. Doering J. Hautaniemi S. Roblick U. J. Bundgen N. K. Nicorici D. (2009). The gene expression signature of genomic instability in breast cancer is an independent predictor of clinical outcome. *Int. J. Cancer* 124 1552–1564. 10.1002/ijc.24017 19101988 PMC2707256

[B17] Hanahan D. Weinberg R. A. (2011). Hallmarks of cancer: the next generation. *Cell* 144 646–674. 10.1016/j.cell.2011.02.013 21376230

[B18] He J. Yu J. (2019). Long noncoding RNA FAM83A-AS1 facilitates hepatocellular carcinoma progression by binding with NOP58 to enhance the mRNA stability of FAM83A. *Biosci. Rep.* 39:BSR20192550. 10.1042/BSR20192550 31696213 PMC6851519

[B19] Hu W. L. Jin L. Xu A. Wang Y. F. Thorne R. F. Zhang X. D. (2018). GUARDIN is a p53-responsive long non-coding RNA that is essential for genomic stability. *Nat. Cell. Biol.* 20 492–502. 10.1038/s41556-018-0066-7 29593331

[B20] Hu X. Feng Y. Zhang D. Zhao S. D. Hu Z. Greshock J. (2014). A functional genomic approach identifies FAL1 as an oncogenic long noncoding RNA that associates with BMI1 and represses p21 expression in cancer. *Cancer Cell* 26 344–357. 10.1016/j.ccr.2014.07.009 25203321 PMC4159613

[B21] Huang G. M. Zang H. L. Geng Y. X. Li Y. H. (2020). LncRNA FAM83A-AS1 aggravates the malignant development of esophageal cancer by binding to miR-495-3p. *Eur. Rev. Med. Pharmacol. Sci.* 24 9408–9415. 10.26355/eurrev_202009_23024 33015782

[B22] Huarte M. (2015). The emerging role of lncRNAs in cancer. *Nat. Med.* 21 1253–1261. 10.1038/nm.3981 26540387

[B23] Iasonos A. Schrag D. Raj G. V. Panageas K. S. (2008). How to build and interpret a nomogram for cancer prognosis. *J. Clin. Oncol.* 26 1364–1370. 10.1200/JCO.2007.12.9791 18323559

[B24] Jachimowicz R. D. Beleggia F. Isensee J. Velpula B. B. Goergens J. Bustos M. A. (2019). UBQLN4 represses homologous recombination and is overexpressed in aggressive tumors. *Cell* 176 505–519e22. 10.1016/j.cell.2018.11.024 30612738

[B25] Jin D. Song Y. Chen Y. Zhang P. (2020). Identification of a seven-lncRNA immune risk signature and construction of a predictive nomogram for lung adenocarcinoma. *Biomed. Res. Int.* 2020:7929132. 10.1155/2020/7929132 32596372 PMC7273488

[B26] Kandoth C. McLellan M. D. Vandin F. Ye K. Niu B. Lu C. (2013). Mutational landscape and significance across 12 major cancer types. *Nature* 502 333–339. 10.1038/nature12634 24132290 PMC3927368

[B27] Kim N. Hong Y. Kwon D. Yoon S. (2013). Somatic mutaome profile in human cancer tissues. *Genomics Inform.* 11 239–244. 10.5808/GI.2013.11.4.239 24465236 PMC3897852

[B28] Kronenwett U. Ploner A. Zetterberg A. Bergh J. Hall P. Auer G. (2006). Genomic instability and prognosis in breast carcinomas. *Cancer Epidemiol. Biomarkers Prev.* 15 1630–1635. 10.1158/1055-9965.EPI-06-0080 16985023

[B29] Lee J. K. Choi Y. L. Kwon M. Park P. J. (2016). Mechanisms and consequences of cancer genome instability: lessons from genome sequencing studies. *Annu. Rev. Pathol.* 11 283–312. 10.1146/annurev-pathol-012615-044446 26907526

[B30] Lee S. Kopp F. Chang T. C. Sataluri A. Chen B. Sivakumar S. (2016). Noncoding RNA NORAD regulates genomic stability by sequestering PUMILIO proteins. *Cell* 164 69–80. 10.1016/j.cell.2015.12.017 26724866 PMC4715682

[B31] Li Z. Xu D. Chen X. Li S. Chan M. T. V. Wu W. K. K. (2020). LINC01133: an emerging tumor-associated long non-coding RNA in tumor and osteosarcoma. *Environ. Sci. Pollut. Res. Int.* 27 32467–32473. 10.1007/s11356-020-09631-1 32556990

[B32] Liao M. Liu Q. Li B. Liao W. Xie W. Zhang Y. (2018). A group of long noncoding RNAs identified by data mining can predict the prognosis of lung adenocarcinoma. *Cancer Sci.* 109 4033–4044. 10.1111/cas.13822 30290038 PMC6272079

[B33] Ling H. Spizzo R. Atlasi Y. Nicoloso M. Shimizu M. Redis R. S. (2013). CCAT2, a novel noncoding RNA mapping to 8q24, underlies metastatic progression and chromosomal instability in colon cancer. *Genome Res.* 23 1446–1461. 10.1101/gr.152942.112 23796952 PMC3759721

[B34] Liu S. Xi X. (2020). LINC01133 contribute to epithelial ovarian cancer metastasis by regulating miR-495-3p/TPD52 axis. *Biochem. Biophys. Res. Commun.* 533 1088–1094. 10.1016/j.bbrc.2020.09.074 33036757

[B35] Lo Y. M. Tsui N. B. Chiu R. W. Lau T. K. Leung T. N. Heung M. M. (2007). Plasma placental RNA allelic ratio permits noninvasive prenatal chromosomal aneuploidy detection. *Nat. Med.* 13 218–223. 10.1038/nm1530 17206148

[B36] Mackay H. L. Moore D. Hall C. Birkbak N. J. Jamal-Hanjani M. Karim S. A. (2018). Genomic instability in mutant p53 cancer cells upon entotic engulfment. *Nat. Commun.* 9:3070. 10.1038/s41467-018-05368-1 30076358 PMC6076230

[B37] Meng L. Xing Z. Guo Z. Liu Z. (2020). LINC01106 post-transcriptionally regulates ELK3 and HOXD8 to promote bladder cancer progression. *Cell Death Dis.* 11:1063. 10.1038/s41419-020-03236-9 33311496 PMC7733594

[B38] Mettu R. K. Wan Y. W. Habermann J. K. Ried T. Guo N. L. (2010). A 12-gene genomic instability signature predicts clinical outcomes in multiple cancer types. *Int. J. Biol Markers* 25 219–228. 10.5301/jbm.2010.6079 21161944 PMC3155635

[B39] Munschauer M. Nguyen C. T. Sirokman K. Hartigan C. R. Hogstrom L. Engreitz J. M. (2018). The NORAD lncRNA assembles a topoisomerase complex critical for genome stability. *Nature* 561 132–136. 10.1038/s41586-018-0453-z 30150775

[B40] Negrini S. Gorgoulis V. G. Halazonetis T. D. (2010). Genomic instability–an evolving hallmark of cancer. *Nat. Rev. Mol. Cell Biol.* 11 220–228. 10.1038/nrm2858 20177397

[B41] Oh J. H. Jang S. J. Kim J. Sohn I. Lee J. Y. Cho E. J. (2020). Spontaneous mutations in the single TTN gene represent high tumor mutation burden. *NPJ Genom. Med.* 5:33. 10.1038/s41525-019-0107-6 32821429 PMC7424531

[B42] Ottini L. Falchetti M. Lupi R. Rizzolo P. Agnese V. Colucci G. (2006). Patterns of genomic instability in gastric cancer: clinical implications and perspectives. *Ann. Oncol.* 17(Suppl. 7) vii97–vii102. 10.1093/annonc/mdl960 16760303

[B43] Polo S. E. Blackford A. N. Chapman J. R. Baskcomb L. Gravel S. Rusch A. (2012). Regulation of DNA-end resection by hnRNPU-like proteins promotes DNA double-strand break signaling and repair. *Mol. Cell* 45 505–516. 10.1016/j.molcel.2011.12.035 22365830 PMC3550743

[B44] Qin N. Wang C. Lu Q. Ma Z. Dai J. Ma H. (2017). Systematic identification of long non-coding RNAs with cancer-testis expression patterns in 14 cancer types. *Oncotarget* 8 94769–94779. 10.18632/oncotarget.21930 29212265 PMC5706911

[B45] Sharma D. Singh A. Pathak M. Kaur L. Kumar V. Roy B. G. (2020). DNA binding and antiradical potential of ethyl pyruvate: key to the DNA radioprotection. *Chem. Biol. Interact.* 332:109313. 10.1016/j.cbi.2020.109313 33171137

[B46] Shi R. Jiao Z. Yu A. Wang T. (2019). Long noncoding antisense RNA FAM83A-AS1 promotes lung cancer cell progression by increasing FAM83A. *J. Cell Biochem.* 120 10505–10512. 10.1002/jcb.28336 30659636 PMC6590457

[B47] Siegel R. L. Miller K. D. Jemal A. (2020). Cancer statistics, 2020. *CA Cancer J. Clin.* 70 7–30. 10.3322/caac.21590 31912902

[B48] Statello L. Guo C. J. Chen L. L. Huarte M. (2020). Gene regulation by long non-coding RNAs and its biological functions. *Nat. Rev. Mol. Cell Biol*. 22 96–118. 10.1038/s41580-020-00315-9 33353982 PMC7754182

[B49] Sun J. Zhang Z. Bao S. Yan C. Hou P. Wu N. (2020). Identification of tumor immune infiltration-associated lncRNAs for improving prognosis and immunotherapy response of patients with non-small cell lung cancer. *J. Immunother. Cancer* 8:e000110. 10.1136/jitc-2019-000110 32041817 PMC7057423

[B50] Suzuki K. Ohnami S. Tanabe C. Sasaki H. Yasuda J. Katai H. (2003). The genomic damage estimated by arbitrarily primed PCR DNA fingerprinting is useful for the prognosis of gastric cancer. *Gastroenterology* 125 1330–1340. 10.1016/j.gastro.2003.07.006 14598249

[B51] Varella-Garcia M. (2010). Chromosomal and genomic changes in lung cancer. *Cell Adh. Migr.* 4 100–106. 10.4161/cam.4.1.10884 20139701 PMC2852566

[B52] Wang T. Wang G. Zhang X. Wu D. Yang L. Wang G. (2017). The expression of miRNAs is associated with tumour genome instability and predicts the outcome of ovarian cancer patients treated with platinum agents. *Sci. Rep.* 7:14736. 10.1038/s41598-017-12259-w 29116111 PMC5677022

[B53] Xiao G. Wang P. Zheng X. Liu D. Sun X. (2019). FAM83A-AS1 promotes lung adenocarcinoma cell migration and invasion by targeting miR-150-5p and modifying MMP14. *Cell Cycle* 18 2972–2985. 10.1080/15384101.2019.1664225 31522616 PMC6791711

[B54] Yang W. Yue Y. Yin F. Qi Z. Guo R. Xu Y. (2020). LINC01133 and LINC01243 are positively correlated with endometrial carcinoma pathogenesis. *Arch. Gynecol. Obstet*. 303 207–215. 10.1007/s00404-020-05791-0 32929617

[B55] Yang Y. Zhang J. Chen Y. Xu R. Zhao Q. Guo W. (2020). MUC4, MUC16, and TTN genes mutation correlated with prognosis, and predicted tumor mutation burden and immunotherapy efficacy in gastric cancer and pan-cancer. *Clin. Transl. Med.* 10 e155. 10.1002/ctm2.155 32898332 PMC7443139

[B56] Zang C. Nie F. Q. Wang Q. Sun M. Li W. He J. (2016). Long non-coding RNA LINC01133 represses KLF2, P21 and E-cadherin transcription through binding with EZH2, LSD1 in non small cell lung cancer. *Oncotarget* 7 11696–11707. 10.18632/oncotarget.7077 26840083 PMC4905504

[B57] Zeng L. Lyu X. Yuan J. Wang W. Zhao N. Liu B. (2020). Long non-coding RNA LINC01116 is overexpressed in lung adenocarcinoma and promotes tumor proliferation and metastasis. *Am. J. Transl. Res.* 12 4302–4313. 32913506 PMC7476163

[B58] Zeng L. Wang W. Chen Y. Lv X. Yuan J. Sun R. (2019). A five-long non-coding RNA signature with the ability to predict overall survival of patients with lung adenocarcinoma. *Exp. Ther. Med.* 18 4852–4864. 10.3892/etm.2019.8138 31777562 PMC6862666

[B59] Zhai X. Wu Y. Wang Z. Zhao D. Li H. Chong T. (2020). Long noncoding RNA LINC01133 promotes the malignant behaviors of renal cell carcinoma by regulating the miR-30b-5p/Rab3D Axis. *Cell Transplant.* 29:963689720964413. 10.1177/0963689720964413 33054325 PMC7784578

[B60] Zhang J. Zhu N. Chen X. (2015). A novel long noncoding RNA LINC01133 is upregulated in lung squamous cell cancer and predicts survival. *Tumour Biol.* 36 7465–7471. 10.1007/s13277-015-3460-9 25908174

[B61] Zhang S. Pan X. Zeng T. Guo W. Gan Z. Zhang Y. H. (2019). Copy number variation pattern for discriminating MACROD2 states of colorectal cancer subtypes. *Front. Bioeng. Biotechnol.* 7:407. 10.3389/fbioe.2019.00407 31921812 PMC6930883

[B62] Zhou M. Guo M. He D. Wang X. Cui Y. Yang H. (2015). A potential signature of eight long non-coding RNAs predicts survival in patients with non-small cell lung cancer. *J. Transl. Med.* 13:231. 10.1186/s12967-015-0556-3 26183581 PMC4504221

[B63] Zhou M. Shao W. Dai H. Zhu X. (2020). A robust signature based on autophagy-associated LncRNAs for predicting prognosis in lung adenocarcinoma. *Biomed. Res. Int.* 2020:3858373. 10.1155/2020/3858373 32190662 PMC7072108

[B64] Zhou M. Xu W. Yue X. Zhao H. Wang Z. Shi H. (2016). Relapse-related long non-coding RNA signature to improve prognosis prediction of lung adenocarcinoma. *Oncotarget* 7 29720–29738. 10.18632/oncotarget.8825 27105492 PMC5045428

