# Supplementary material for: Identification of the Prognostic Significance of Somatic Mutation-Derived LncRNA Signatures of Genomic Instability in Lung Adenocarcinoma
Source: Front Cell Dev Biol. 2021 Mar 29;9:657667. doi: 10.3389/fcell.2021.657667 (PMC8039462; doi:10.3389/fcell.2021.657667)
Supplement: Supplementary file 1 [file Data_Sheet_1.zip › Supplementary Figures and Tables/Captions of Supplementary Figures and Tables.docx]

**Supplementary Figures and Tables**

**Supplementary Figures**

**Supplementary Figure S1.** Calibration assessment of the nomogram. (A) Calibration plot of the nomogram in the training set (A), testing set (B), and TCGA set (C), respectively.

**Supplementary Tables**

**Supplementary Table S1.** Clinical information of patients with LUAD in GSL and GUL group.

**Supplementary Table S2.** Differentially expressed lncRNAs between the GUL group and the GSL group.

**Supplementary Table S3.** Go enrichment analyses of the co-expressed protein genes with GInLncRNAs.

**Supplementary Table S4.** Screening of eleven GInLncRNAa associated with LUAD patients' survival using univariate Cox regression analysis.

**Supplementary Table S5.** Screening of seven GInLncRNAa associated with LUAD patients' survival using multivariate Cox regression analysis.

**Supplementary Table S6.** Grouping information of high- and low-risk groups based on the GInLncSig score in the training set.

**Supplementary Table S7.** Grouping information of high- and low-risk groups based on the GInLncSig score in the testing set.

**Supplementary Table S8.** Grouping information of high- and low-risk groups based on the GInLncSig score in the entire TCGA set.
