# Supplementary figures and images for: Identification of the Prognostic Significance of Somatic Mutation-Derived LncRNA Signatures of Genomic Instability in Lung Adenocarcinoma
Source: Front Cell Dev Biol. 2021 Mar 29;9:657667. doi: 10.3389/fcell.2021.657667 (PMC8039462; doi:10.3389/fcell.2021.657667)

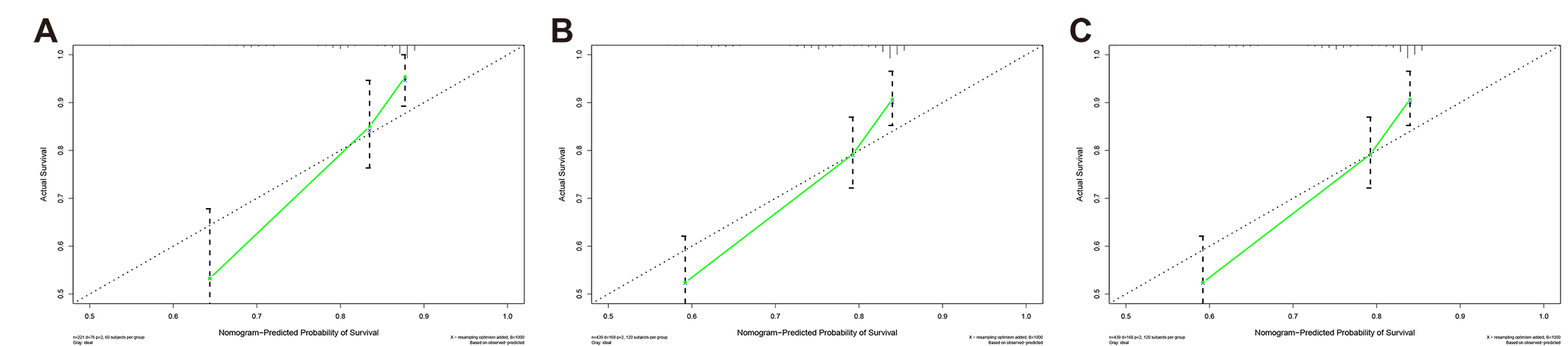

Supplement: Supplementary file 1 [file Data_Sheet_1.zip › Supplementary Figures and Tables/Figure S1.tif]
